# Renewable Carbon Nanomaterials: Novel Resources for Dental Tissue Engineering

**DOI:** 10.3390/nano11112800

**Published:** 2021-10-22

**Authors:** Seyyed Mojtaba Mousavi, Khadije Yousefi, Seyyed Alireza Hashemi, Marzie Afsa, Sonia BahranI, Ahmad Gholami, Yasmin Ghahramani, Ali Alizadeh, Wei-Hung Chiang

**Affiliations:** 1 Department of Chemical Engineering, National Taiwan University of Science and Technology, Taipei 10617, Taiwan; mousavi.nano@gmail.com; 2 Biotechnology Research Center, Shiraz University of Medical Sciences, Shiraz 71348-14336, Iran; khadije.yousefi@gmail.com (K.Y.); marzieafsa@gmail.com (M.A.); 3 Department of Dental Materials and Biomaterials Research Centre, Shiraz Dental School, Shiraz University of Medical Sciences, Shiraz 71345-1583, Iran; 4 Nanomaterials and Polymer Nanocomposites Laboratory, School of Engineering, University of British Columbia, Kelowna, BC V1V 1V7, Canada; sa_hashemi@sums.ac.ir; 5 Pharmaceutical Science Research Center, Shiraz University of Medical Sciences, Shiraz 71348-14336, Iran; S.bahrani22@gmail.com; 6 Department of Endodontics, School of Dentistry, Shiraz University of Medical Sciences, Shiraz 71348-14336, Iran; 7 Department of Tissue Engineering and Applied Cell Sciences, School of Advanced Medical Sciences and Technologies, Shiraz University of Medical Sciences, Shiraz 71345-1583, Iran; alializadeha@gmail.com

**Keywords:** graphene, carbon nanotube (CNT), nanomaterials, tissue engineering

## Abstract

Dental tissue engineering (TE) is undergoing significant modifications in dental treatments. TE is based on a triad of stem cells, signaling molecules, and scaffolds that must be understood and calibrated with particular attention to specific dental sectors. Renewable and eco-friendly carbon-based nanomaterials (CBMs), including graphene (G), graphene oxide (GO), reduced graphene oxide (rGO), graphene quantum dots (GQD), carbon nanotube (CNT), MXenes and carbide, have extraordinary physical, chemical, and biological properties. In addition to having high surface area and mechanical strength, CBMs have greatly influenced dental and biomedical applications. The current study aims to explore the application of CBMs for dental tissue engineering. CBMs are generally shown to have remarkable properties, due to various functional groups that make them ideal materials for biomedical applications, such as dental tissue engineering.

## 1. Introduction

Tissue engineering (TE) and nanomaterials (NM) in dentistry have modified perspectives and medical actions. Since the turn of the century, autologous grafts or autologous cloth tissues, heterologous substances for bone or mucous losses, and heterologous biocompatible substances have all been used to update and repair oromaxillofacial misplaced tissues for traumas or different diseases (amalgam, composite resins, glass ionomer cement, and gutta-percha) withinside the partial lack of dental tissues or, additionally, the usage of osseointegrate substances in implant dentistry for the whole replacement of the misplaced tooth in total [1]. From 1980 to 1990 [2,3], new membranes emerged with the potential to improve guided tissue regeneration (GTR) in dentistry, and autologous platelet concentrates (PRP and PRF) with membranes for restoration. Guided bone loss in oral surgery can be considered the beginning of new techniques based on three main elements of tissue engineering: stem cells, media, and signaling molecules [2].

NM and TE have been incorporated into dentistry in recent decades by introducing nanotechnologies in the composition of scaffold matrices (rigid and soft), growth factors, and stem cells, and the development of biomodulation techniques for dental tissue reconstruction. Carbon-based nanomaterials are now in the spotlight of biomedical research. They have established a prime position, ranging from drug delivery to tissue engineering [3,4]. Biomass-based carbon quantum dots have become substantial carbon materials because of their cost effectiveness, ease of fabrication, and lower environmental impact. Graphene and carbon nanotubes(CNTs) are among the nano carbon-based materials used in biomedical and clinical research, due to their unique characteristics, including low toxicity, high solubility, strong inertness, high specific surface areas, abundant edge sites, and versatility [5]. Hence, the addition of graphene to biomaterials has provided scientists with a broad spectrum of materials that can be manipulated for various purposes. Although cytotoxicity is influenced by multiple factors, such as shape, concentration, size, and applied dosage [4,5], it is of major interest to manipulate graphene-based materials to maximize their biocompatibility and minimize their cytotoxicity. The introduction of graphene quantum dots (GQD) has, to some extent, overcome the shortcomings of graphene in biomedical applications. Besides lowering the cytotoxic effects of graphene, graphene quantum dots have shown sustainable antibacterial properties against Gram-negative and Gram-positive dental pathogens [6].

Furthermore, recent studies have reported that CNTs has prior efficiency in treating dentin hypersensitivity [7] and mesenchymal stem cell differentiation [8,9]. However, there is still debate on the potential applications of this derivative for orthopedic dedications. Surface functionalization of graphene-based nanomaterials (GBnMs) with diverse bioactive and bioinert molecules facilitates their extensive application for implant and bone scaffold improvements [10], subsequently increasing the potential of bone integration and long-term implant success. Therefore, this review study aims to survey the applications, advantages, and disadvantages of eco-friendly and renewable GBnMs in implantology and scaffold improvements.

## 2. Tissue Engineering in Dentistry

**Figure 1:**
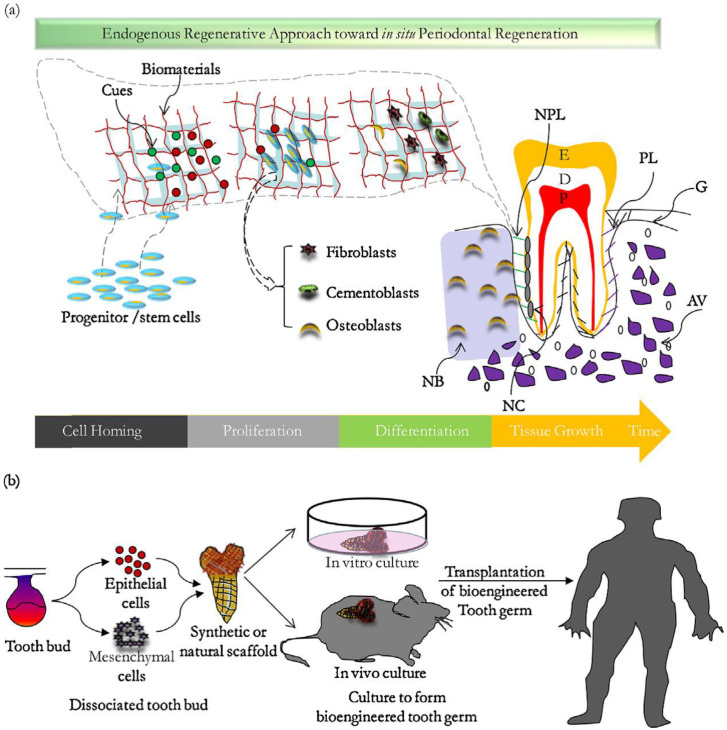
(**a**) Schematic diagram of the endogenous method for periodontal tissue regeneration. E: enamel, D: dentin, P: pulp, G: gum, PL: periodontal ligament, AB: alveolar bone, NPL: new periodontal ligament, NB: new bone, NC: new bone cement. (**b**) Strategies for designing complete teeth [16].

In addition to conventional therapies, directed tissue engineering methods for periodontal disease resolution have shown considerable growth. However, reproducing the unique structure and function of the whole tooth and periodontal system remains challenging. Despite some minor accomplishments, developing repeatable, clinically safe oral tissue repair and regeneration procedures faces substantial hurdles. There is significant evidence to support the necessity of this treatment, and worldwide public health data indicate that patient capacity is more than enough. The connective tissues that support the teeth (gingiva, alveolar bone, periodontal ligament, and root canal) are destroyed by gingival inflammation, resulting in tooth loss (Figure 1a) [11].

The tooth support structure, namely the simentuzil periodontal ligament bone contact and structure, is complicated to regenerate. The GTR/GBR membrane (guided tissue/bone regeneration membrane) utilizes an obstructive membrane to preserve a deficiency, regenerate tissue that has lost suitable cells, and sustain newly created tissue [12]. Carbon nanotubes (CNTs) and carbon nanotube-based composites (CNTs combined with other polymers) were recently found to be promising biomaterials for dental tissue regeneration. Martins Junior et al. [13] provides an excellent overview of bone tissue engineering, focusing on the potential effects of CNTs in bone formation and recovery/regeneration. Combinations of synthetic and natural polymers can make nanofiber scaffolds with various properties. Endogenous regeneration (ERT) is a method that uses significant endogenous resources (such as cells, growth hormones, and proteins) to regenerate functioning tissues (Figure 1b). Cell return, also known as cell transplantation, is a promising method for complete and consistent periodontal repair that is cell dependent [14]. Clinical results are influenced by the choice and design of each component and the invasiveness of the clinical process. In the event of cell return, a gap between substances is necessary to attract host stem cells to regenerate periodontal disease (for example, growth factors attached to fibrin, Emdogain, and BioOss) [15].

## 3. Graphene-Based Nanostructures

Carbon nanostructures are divided into four categories: zero-, one-, two-, and three-dimensional structures. The graphene derivatives (e.g., graphene and its oxidized and reduced forms) are considered two-dimensional carbon structures that present remarkable characteristics [17]. Graphene is a hexagonal honeycomb matrix consisting of firmly packed Sp^2^ hybridized carbon atoms [18] that can act as a precursor to various structures, such as graphene nanotubes, nanoribbons, and quantum dots.

Supreme material parameters, such as the unique mechanical resistance, low cytotoxicity, especially osteoblasts, and intrinsic antibacterial activity [19,20], have made graphene a forefront material for bone tissue engineering. Graphene might have the potential to replace other materials in tissue engineering, owing to the mentioned properties. Graphene can be functionalized [21] with small molecules, nanoparticles, and polymers via chemical interactions [22]. The emergence of different devices that can meet specific characteristics for various purposes is dedicated to the ability of graphene surfaces to be functionalized with molecules of diverse nature [10,23,24].

**Figure 2:**
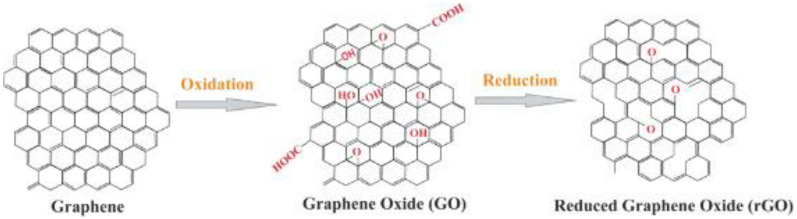
The conversion process of graphene to GO and rGO [28].

The oxidized form of graphene, GO, contains many oxygen-containing functional groups (i.e., hydroxyl groups and epoxies on the basal plane and carboxyl groups on the edges) while preserving the same thin atom structure graphene [25]. The presence of oxygen-containing functionalities provides GO with more active sites than graphene, facilitating the covalent or noncovalent interaction with biomolecules and other nanomaterials [26,27]. When reduction occurs through chemical, thermal, or other approaches, the oxygen functionalities (i.e., carboxyl, epoxide, carbonyl, and carbonyl groups) of rGO become far fewer in contrast to GO; yet, it still contains a particular extent of reactive oxygen, the quantity of which is determined by the reduction methods and conditions (Figure 2).

One of the old drawbacks of using graphene and its derivatives in industries is the relatively high price of highly pure graphene on an industrial scale. Although this problem is less common in biomedical uses, it could make it less applicable. Several methods for preparing these high purity materials have been developed at affordable prices on the industrial scale. The ability to produce graphene from low-cost carbon sources, such as coal, petroleum coke, biochar, carbon black, discarded food, rubber tires, and mixed plastic waste, deducts costs through the manufacturing process. The price of raw graphite used in the exfoliation process is estimated at USD 1000 to USD 3500 per ton, while coal costs USD 25 per ton. Luong and co-workers successfully developed a unique strategy for high-quality graphene synthesis named “flash Joule heating,” in which household waste carbon materials are instantly converted into high pure crystalline graphene [29]. They produced a high yield (over 90%) of highly pure graphene, a valuable technique for industrial-scale synthesis that is both cost-effective and sustainable. Interestingly, this method does not require reactive gases, solvents, or furnaces, and it produces a product with a purity of over 99% without any purification step. The amount of electric energy used to prepare this type of graphene is only 7.2 kj/g, making it suitable for nanocomposites.

That this technique provides us with high-quality graphene is the reason for rapid progress in graphene research. Recently, various approaches have been developed to fabricate eco-friendly CDs; the reduction in graphene oxide (GO) using nature-based reagents and eco-friendly methods has attracted a great deal of scientific interest-targeted ultimately at the bulk scale production of graphene for its commercial applications. There are many natural antioxidants, including amino acids, vitamins and organic acids, which are readily used to reduce GO [30].

Different plant extracts are applied as the reducing agents for GO, as they contain polyphenolic compounds that are readily oxidizable and become converted to the corresponding quinone forms. The reduction abilities of such phytochemicals were observed previously in the synthesis and stabilization of Au, Ag, Pd, and Fe nanoparticles [31].

Although different plant extracts have been explored as reducing agents, their basic reduction mechanisms are the same. Plant extract contains plentiful polyphenols, which have a high tendency to oxidize. Polyphenol reacts with the epoxide moiety through an SN^2^ mechanism, resulting in the opening of the oxirane ring. The carbonyl and hydroxyl groups experience similar nucleophilic attacks by polyphenol with the elimination of a water molecule. This results in the successful conversion of GO to rGO [32]. The use of green reductants in the chemical reduction of GO has proven to be environmentally friendly, and the product obtained is highly dispersible and biocompatible [33].

There are three primary techniques for graphene synthesis, including chemical vapor deposition, exfoliation, and chemical-based techniques [34]. Among these, industrial-scale production is highly possible through solution-based chemical reduction of GO. Natural graphene is oxidized to graphite oxide, then exfoliated by sonication to GO, followed by reduction to produce reduced graphene oxide (rGO). The binding of GO and rGO to dexamethasone was mainly studied by Na Ren et al. [35]. They observed that samples coated with DEX-GO absorbed the most DEX and sustainably released it through time. DEX release represented a linear sample at early stages and gradually reduced over time, due to material depletion. Since the presence of oxygen-containing groups and electrostatic interactions of GO contributed to the improved wettability and therefore biocompatibility of the composite, the proliferation of rBMSCs was promoted in this study, with DEX-GO-Ti showing the highest filopodial extension. In addition, ALP activity showed that DEX-GO-Ti and DEX-rGO-Ti both increased the proliferation of rBMSCs. Wettability is a crucial factor in biomedical treatments that necessitate cell-biomaterials interactions because the level of surface wettability considerably affects various biological events. Wettability is a determining factor in adsorption and adhesion phenomena that are fundamental for cell adhesion and proliferation to occur [36]. The sustained release of biomolecules was also confirmed by Liping Ren et al. [37]. Their results showed a 3-day sustained release of aspirin when loaded on GO-coated Ti, which they referred to as a result of the interaction of benzene rings in aspirin and functional groups of GO, as well as π–π stacking interactions. Differences in the pace and level of aspirin release among test groups of this study (A/Ti, A/Ti-GO) are attributed to different levels of osseointegration in MC3T3-1E cells. Generally, the increased biocompatibility of Ti surfaces due to coating with graphene was confirmed by many authors that investigated the in vitro biological properties of graphene and its derivatives on various cell lines. GO coatings were efficiently performed in in vivo studies as well. The use of GO-coated Ti as a substrate for delivering BMP-2 to the mouse model calvarial defects showed that the loading of BMP-2 on GO-Ti enables the deposition of large doses and sustainable release along with preservation and bioactivity of the biomolecule [38].

However, there is still debate on the cytotoxicity of graphene-based materials in mammalian cells. Li et al. [39] evaluated the toxicity of hydrated graphene, pristine GO, and rGO in murine lung macrophages. Despite hydrated graphene being in charge of the highest radical carbon density and cell death in TPH-1 and BEAS-2B, pristine GO also showed toxic effects on the cells to some extent, while rGO presented minimal effects. It is anticipated that the surface oxidation state and carbon radical content play significant roles in inducing toxicity by GO in mammalian lung cells. Nonetheless, the emergence of green graphene production methods will hopefully expand horizons in the application of GBMs. For example, the in vivo application of green rGO-coated Ti in exposure to rat osteoblasts showed superior biocompatibility and promotion in cell adhesion and proliferation and did not represent any toxicity on the target cells [40].

Another acute complication in the utilization of dental implants is the formation of biofilms and the accumulation of primary agents on the implant’s surface. Various coatings with enhanced antibacterial properties are suggested to overcome the challenge. Not only can GBnMs be used as coatings per se, but the incorporation of other antibacterial nanomaterials, such as Ag, Zn, etc., will improve the antibacterial properties of implant coatings. An investigation assessed graphene-based coating as incorporating non-toxic zinc oxide to the graphene coating on an artificial acrylic teeth surfaces nanocomposite and observed a substantial decrease in the deposition of the carcinogenic *S. mutans* bacteria and biofilm formation [41].

The combination of GO-coating and brushing is also believed to promote the osteogenesis of contaminated Ti since effective mechanical removal and the antibacterial activity of GO at a concentration of 256 µg/mL causes precipitation of GO nanosheets on the surface of Ti and enhances osteogenic differentiation [42]. Nevertheless, GO was less toxic to *S. mutans* than other dental biofilm pathogens, such as *P. gingivalis* and *F. nucleutum*. GBnMs have been developed as valuable agents that can be utilized in a broad spectrum of biomedical applications. The existing data in the literature would seem to confirm that conventional biomaterials can be well characterized for better performance in living tissues. Yet, it must be noted that the GBnM-incorporated materials’ behavior is thoroughly related to the intrinsic properties of graphene, such as its physicochemical properties, size, concentration, structural additives, and surface functionalization [10].

### 3.1. Biocompatibility and Cytotoxicity of Graphene-Based Materials

The fundamental feature of a newly developed nanomaterial for use in biomedical applications is its biocompatibility. Supreme biocompatibility is a vital aspect of material for avoiding deleterious effects in living tissues. GBMs are instantaneously growing in biomedical applications; therefore, assessments of their biocompatibility and cytotoxicity are necessary. When it comes to biomedical and pharmaceutical applications, acquiring biocompatible graphene is more critical. To be utilized in dentistry, considering several factors in biocompatibility is essential. First, it must be compatible with oral fluids and not release toxic products into the oral cavity. It must also have sufficient strength and durability to meet the expectations [43]. Furthermore, the material must prevent the production of biofilms and bacterial growth on the scaffold to which it is put. In general, preventing an unfavorable effect inside live tissues and ensuring that the material can lead to effective tissue engineering procedures are essential elements that provide the material’s biocompatibility success.

Reports from previous studies propose that the cytotoxicity of GBnMs is determined by various factors, such as their concentration, shape, size, dispersibility, and surface functionalization. MTT assays used in in vitro studies show that among different shapes of few-layer graphene sheets (FLGS) and single wall carbon nanotubes (SWCNT), FLGSs have higher toxicity than SWCNTs at low concentrations. Moreover, they are less toxic at higher concentrations of G. In addition, it confirms the idea that MTT assays show that FLGSs are more toxic than SWCNTs at low concentrations and less toxic at higher concentrations of G sheets in a solution. As a result, it can interfere with cell viability by inducing oxidative stress in the cell membrane, penetration through the membrane and causing physical impairment and hydrophobic interactions between graphene and cell lipids. Similarly, GO in solution increases the formation of mitochondrial reactive oxygen species and induces cell death in mouse alveolar macrophages. On the contrary, it cannot reduce human cellular proliferation either as a film or in combination with other biomaterials.

**Figure 3:**
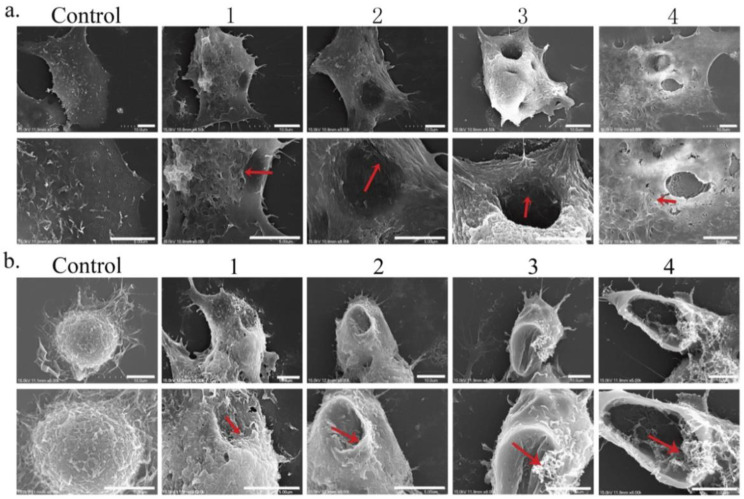
SEM images of A549 (**a**) and Raw264.7 (**b**) cells undergoing cell membrane degradation as a result of GO exposure (in its ultimate stage, >24 h). (**b**) Images 1, 2, 3, and 4 reflect different degrees of membrane stress detected during different incubation stages [48].

The addition of various reducing agents to the structure of graphene plays a significant role in modifying its cytotoxicity. For example, studies represent high oxidative stress due to pristine graphene apposition on the cell membrane, while the cell uptake of carboxyl-functionalized graphene was shown to be nontoxic. Various concentrations of GBnMs manifest different levels of cytotoxicity in contact with discrete cell lines, signifying the importance of cell type on the cytotoxicity of GBnMs. Taken together, the results of previous studies illustrate that graphene and GO are safe for most of the cell lines at a concentration of 50 μg/mL, while rGO can be added to the cell medium at up to 60 μg/mL [44]. A comprehensive toxicology study estimated the toxicity of GO nanoribbons with a coating of (1,2-distearoyl-*sn*-glycero-3-phosphoethanolamine-*N*-[amino(polyethylene glycol)]) (O-GNRs-PEG-DSPE) in HeLa, MCF-7, SKBR3, and NIH3T3 cell lines, using six different biochemical and cellular assays. The results illustrated a dose- and time-dependent manner for the toxicology of O-GNRs-PEG-DSPE in contact with the four cell lines. Among these, the coating seemed to be greatly toxic to HeLa cells, compared to other cell lines [45]. The dose-dependent toxicity of GO was also confirmed by Lammel et al. [46], who observed loss of plasma membrane integrity, due to intense GO-phospholipid interactions. Furthermore, Chang et al. stated that although the uptake of GO (hence, changes in viability, integrity, and mortality of the cells) was not observed and it was non-toxic to the tested cell line (A549), GO induced dose- and size-dependent oxidative stress [47]. Duan et al. [48] clearly showed that GO could induce pore formation on the A549 and Raw264.7 cells, due to cooperative lipid extraction (Figure 3).

Nevertheless, the comparison of GO and rGO has revealed controversial results. Many inconclusive statements are available in the literature regarding the relative cytocompatibility of GO and rGO. Some scientists believe that the maintenance of GO solubility and its controlled reduction makes it possible to be utilized in biomedical applications (including dentistry), given that its toxicity can be greatly minimized [49]. The potential cytotoxicity of GBnMs is markedly dependent on their functionalization degree, as represented in the study of Das et al. [50]. When comparing the toxicity of GO and rGO on the cell lines utilized in the study, the first was shown to be more hazardous than rGO of the same size. When compared to rGO, the authors of this study predicted that ROS production in cells and the presence of more reactive oxygen-containing groups in GO would result in a larger potential for interacting with biological macromolecules and causing cytotoxicity. It was demonstrated that polymer-functionalized rGO, with its high water solubility, has an excellent cytocompatibility toward endothelial cells, even at high concentrations of up to 100 μg/mL [51].

On the other hand, Wu et al. [52] stated that the reduction of GO in the loss of oxygen functionalities leads to substantial morphological changes (polygonal curl-shaped rGO). They revealed in their study that rGO was more toxic to primary bone marrow macrophages than pristine GO. Further, the evaluation of cytotoxic effects of GO and rGO on glioma tumor cells in vitro and in vivo have demonstrated that both these GBnMs enter the cells and cause dose-dependent cytotoxicity, yet rGO was shown to have more toxicity on the tested cell lines [53].

The chemical synthesis of rGO aggravates the toxicity associated with them. So, the implementation of green synthetic methods might minimize the toxicity related to soluble solid graphene since the use of toxic reducing agents can be eliminated [54]. In a recent study, the rGO obtained from *Euphoria heterophylla* extracts was shown to be remarkably cytotoxic against A549 and HepG2 cancer cells. rGO in cancer cell lines caused irregular morphological changes [55]. Investigating the impact of green synthesis of GBnMs, Shubha et al. evaluated the cytotoxicity of GO and green reduced GO (GRGO). The extract of *Ocimum sanctum* was used as a reducing agent in their study. Their results indicated a significantly lower hemolysis rate and inhibition of cell growth for GRGO, compared to GO [56].

Furthermore, reducing GO using *Lantana camara* leaf components that naturally bind to rGO nanosheets increased the antioxidant levels against DPPH free radicals. Moreover, MTT test findings revealed that rGO had improved cytotoxic effects against A549 tumor cell lines [57].

In conclusion, the use of green methods in synthesizing GBnMs has demonstrated the potential to alleviate the biological and environmental constraints associated with their use. Similar to in vitro experiments, in vivo studies also represented a concentration-dependent manner in the cytotoxicity of GBnMs. In addition, scientists must consider other vital factors that determine and manipulate the biological characteristics of GBnMs, exposure time, physicochemical properties, administration route as well as the characteristics of the animals used [58].

### 3.2. Anti-Bacterial Activity of Graphene-Based Nanomaterials

One of the significant complications that occur during biomaterial implantation procedures is infection. The inevitable incidence of antibiotic resistance and adverse effects of these compounds on pathogens and bacteria has led to the emergence of nanomaterials with anti-microbial properties over recent years [59]. Currently, most clinical dental materials lack antibacterial activities, and secondary bacterial infections and biofilm formation are the critical factors of treatment failure in clinical dentistry [60].

The antibacterial property of graphene and GBnMs is dependent on administration factors, such as concentration, exposure time, physicochemical characteristics, and the type of bacteria employed in the studies, just as other graphene features. Furthermore, various variables can alter the outcome of GBnMs’ antibacterial activities in the oral cavity. The most significant variables are host vulnerability, diets, and behaviors that might disrupt the oral microenvironment [61].

**Figure 4:**
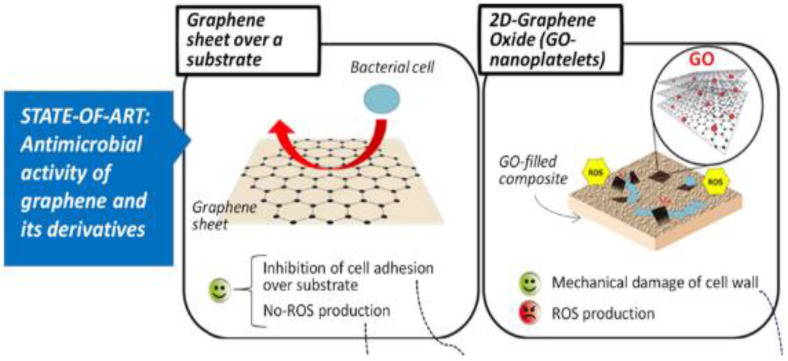
Graphene and GO have antimicrobial and antibiofilm properties, due to their structure and the presence of oxygen-containing functional groups that improve hydrophilicity and facilitate ROS generation [66].

Predominantly, GBnMs, which are smaller in size and have higher amounts of oxygen functionalities incorporated into their structure, can potentially cause more interactions with bacterial cells and hence, more elevated accumulation levels. Direct contact of GBnMs with cells triggers stress in the cell membrane, resulting in cell death [62]. Among carbon-based materials that have so far been discussed, GO showed the most potent antibacterial activity under similar concentrations and incubation conditions, followed by rGO, graphite, and graphite oxide [63]. The antibacterial activity of GBnMs was confirmed previously by many authors. These materials present a significant antibacterial effect against both Gram-positive and Gram-negative bacteria. GBnMs perform their antibacterial properties by breaking down the physical integrity of the bacterial structure (Figure 4). This purpose is maintained via different mechanisms.

Through ROS generation and charge transfer phenomena, GBnMs can act as nano-knifes that penetrate and cut the cell membrane, causing mechanical and oxidative stress. According to molecular dynamic simulations, thin graphene nanosheets permanently damage the bacterial membrane due to Van der Waals and hydrophobic interactions of graphene with the bacterial cell’s lipid layer.

GO nanostructures can promptly kill dental pathogens, as was confirmed via TEM images. As novel forms of antibacterial agents, GO nanostructures have several advantages over classic antibiotics since they reduce the potential of the emergence of drug resistance and have a notably lower cost for their wide range of applications. However, the oral cavity is a complex network that includes teeth, root canals, mucosa, periodontal tissues, saliva, and dentures. Mouth bacterial biofilms can form on either side of the oral cavity and contain bacteria and the chemicals they create or collect [64]. It was observed that pure GO nanostructures have toxic effects on the growth of mature biofilms from individual bacterial cells [65].

Bacterial biofilm has high pathogenicity because it is less sensitive to drugs but more resistant to physical assault [67,68]; this explains why GO nanostructures are effective at killing planktonic pathogens and preventing bacterial biofilm formation. Although GO has a preventative impact on the production of Gram-positive and Gram-negative bacteria biofilms at high concentrations, low GO concentrations can stimulate the creation of these biofilms, resulting in an opposite response to the intended effect [69]. It was observed by Pang et al. that both the cytocompatibility and antibacterial activity of GBnMs are dose dependent. They suggested that GO should be administered at a concentration of 50–100 µg/mL to maintain cytotoxicity at the minimum level while maximizing antibacterial activity.

**Figure 5:**
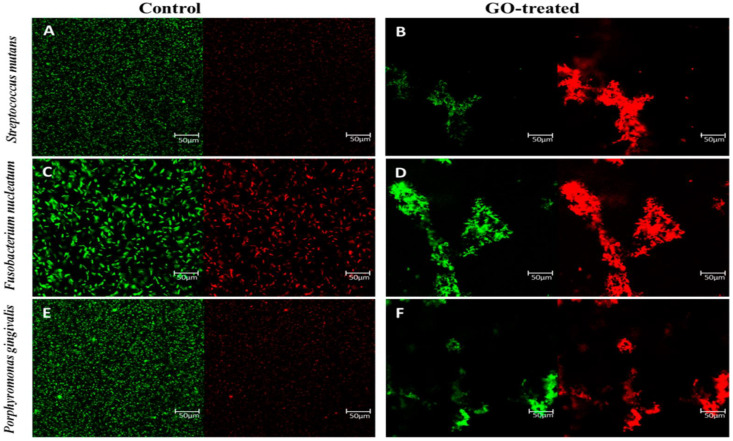
Images of fluorescent stains that are both alive and dead. *S. nutans* (**A**–**D**), *F. mutans*, and *P. gingivalis* cells (**E**,**F**) were treated for 2 h with GO nanosheets and isotonic saline (control). Representative fluorescence microscopy pictures of bacteria cells stained for 15 min in the dark with SYTO 9 (green channel) and PI (red channel). The identical GO dosage of 80 g/mL was given to all of the patients. The scale bar is 50 μm [70].

He et al., in an investigation of the effect of GO against dental pathogen bacteria, observed that the exposure of *S. mutans, P*. *gingivalis*, and *F. nucleatum* to GO nanosheets significantly reduced the viability of these pathogens in a dose-dependent attitude. The bacterial growth of *P. gingivalis* and *F. nucleatum* was shown to be repressed at a GO concentration of 40 µg/mL. At the same time, this amount of GO had limited effects on the viability of *S. mutans*. The experiment showed that a concentration as high as 80 µg/mL was capable of the absolute elimination of *S. mutans*. The TEM images showed that, in the presence of GO, the integrity of dental pathogens was significantly jeopardized, due to the effects mentioned above of GO on the physical integrity of the cell phospholipid membrane (Figure 5) [70].

On the contrary, in another study, GO showed an excellent antibacterial effect on *S. mutans* in both planktonic and biofilm forms in a concentration-dependent manner. In addition, GO nanosheets with more oxygen-containing functional groups showed higher toxicity at low concentrations, implying that the functional groups played a dominant role in the antibacterial outcome. Given that *S. mutans*, as the main etiological factor for dental caries, plays a physiological role in cariogenic biofilms, the findings that GO aqueous dispersions can eradicate biofilms at a relatively low dose (80 µg/mL) are of great importance, regarding their dental applications [60].

Patil et al. incorporated Ag nanoparticles into green synthesized graphene nanocomposites and compared the minimum inhibitory concentrations of GO and rGO when combined with Ag NPs [71]. They effectually synthesized rGO/AgNCs by using sapodilla peel extract as a reducing agent under sunlight irradiation. The obtained rGO/AgNCs unveiled the highest MIC of 7.81 μg/mL against *Pseudomonas aeruginosa*, while the smallest MIC of 15.62 μg/mL was observed against *Staphyloccous aureus*. GO exhibited the highest MIC of 62. μg/mL against *Pseudomonas aeruginosa* and *Staphyloccous aureus*. Accordingly, rGO/AgNCs might be supreme antibacterial agents to prevent dental disorders [72]. In previous research, rGO was decreased by *Lantana camara* extract. It also had the best bactericidal effects against *B*. *subtilis* bacterial pathogens, such as *E. coli* [57].

## 4. Applications of Graphene and Its Derivatives in the Tissue of Dentistry

To restore and regenerate the injured tissues and organs are the objectives of tissue engineering [73,74]. To obtain successful bone tissue engineering, the degree of functionality of the scaffold is a crucial factor. Consequently, the discovery of novel materials for scaffolds, owing to characteristics such as acceptable biocompatibility, disciplined nontoxic degradation, capability for supporting cell differentiation, growth, proliferation, and proper mechanical strength, is essential for the effectiveness of tissue engineering [75,76,77]. Recently, graphene and its derivatives were studied as a novel option for tissue engineering in dentistry, due to their exceptional mechanical properties, stiffness, and electrical conductivity [73]. The effectiveness of mesoporous bioactive glass nanoparticle (MBN)/graphene oxide (GO) composites on the mineralization potency and human dental pulp stem cells differentiation was investigated in research (hDPSCs). It was claimed that MBN/GO composites can help hDPSCs differentiate into odontoblast-like cells and perhaps stimulate dentin production. Thus, they might be used in dentin-pulp complex tissue engineering. MBN-GO composites stimulate mineralization, a key process for dentin regeneration, by increasing odontogenic differentiation markers’ mRNA and protein expression in hDPSCs. The Wnt/ß-catenin signaling system was enhanced by the MBN/GO composites, which promoted odontogenic differentiation [78]. In other words, graphene promotes bone formation by increasing the production of the ß-catenin protein [79]. Another study looked at the biocompatibility of GO-coated collagen membranes on human dental pulp stem cells, emphasizing biomaterial cytotoxicity, and the capacity to promote DPSC development and control inflammation induction.

**Figure 6:**
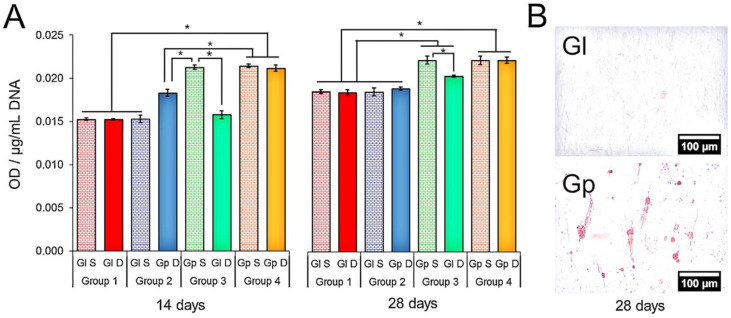
(**A**) Alizarin red S staining. The media obtained from DPSC on Gp increased the mineralization of cells on Gl (Group 3) after 28 days (* denotes statistical difference between the groups, *p* < 0.05). (**B**) Alizarin red S staining evinced the presence of calcium-rich deposits in the DPSCs cultured on Gp (28 days) [81].

Compared to frequently utilized GBR membranes, GO-coated membranes can boost and fulfill osteoblastic differentiation while also successfully controlling the occurrence of inflammatory events. Furthermore, the efficiency of GO (10 g/mL) is dose dependent since it is more efficient at higher concentrations; finally, it was concluded that all of these features make GO-coated collagen membranes a good alternative to frequently used membranes, suggesting more efficient bone production in GBR, therefore providing an ideal starting point to investigate clinical performance [80]. Another study looked at graphene’s ability to produce odontogenic and osteogenic development in dental pulp stem cells (DPSC). Graphene made greater mineralization than glass after 14 and 28 days, as seen in Figure 6 (Gl). Without using a chemical persuader, graphene allowed DPSC to differentiate into osteogenic rather than odontoblastic cells. The ability of graphene to be used as a precursor in craniofacial bone tissue engineering research was demonstrated [81].

**Figure 7:**
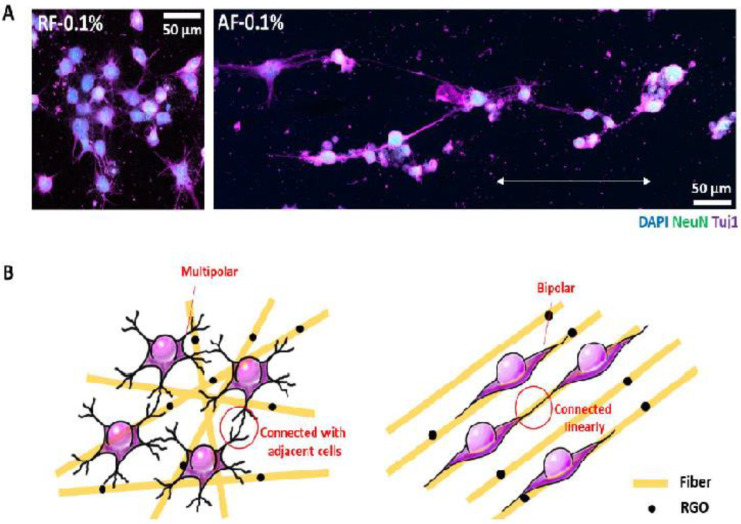
The effect of RGOPCL NF comparison on the differentiation of DPSC. Compare neurites (**A**). The neurites differentiated from 0.1% of RF cells are combined with adjacent cells, but the differentiated neurites of the AF0.1% spread and connect in the direction of the AF arrangement. (**B**) The ideas of RF0.1 in research will be used to create a multi-directional neural network because it can connect differentiated cells with neighboring cells. However, because it can align and link differentiated cells in the direction of AF alignment, AF0.1% can be used to generate unidirectional neural networks [83].

Another study recommended that the admixture of graphene oxide and PDLSCs may be an ideal concept in tissue engineering [82]. According to separate research, a graphene oxide–based substrate facilitated DPSC adhesion, proliferation, and increased the expression of many overexpressed genes in mineral-producing cells. Improved DPSC neurogenesis was achieved by using a suitable concentration of reduced graphene oxide. Furthermore, using random nanofibers improved differentiated cell contiguous connections, but using aligned nanofibers increased differentiated cell junctions across nanofiber alignment directions (as shown in Figure 7). [83]. Another study confirmed the biocompatible characteristics of graphene oxide and its enhanced capacity to produce bone. Tissue engineering is said to benefit from the graphene oxide matrix. Compared to conventional scaffolds, the creation of new bone was enhanced fivefold, according to this study [84]. A recent study found that graphene dental materials had no toxicity toward dental cells after 24 h, as well as no signs of acute cytotoxicity or local inflammation.

## 5. Carbon Nanotube

Carbon nanotubes are carbon cylinders with a wall diameter of nanometers. These pipes, which have open or closed ends, are single-walled or multi-walled. For the synthesis of these tubes, the process of chemical vapor condensation is used. This method involves the decomposition of gases. Hydrocarbons are intermediates alongside metal catalysts. The use of fluidized bed reactors causes uniform penetration of gas and heat into the particles, and in this method, single-walled nanotubes are made. These pipes can also be produced with two other methods of electrical discharge and laser erosion. In the electrical discharge method, a current passes between the gaseous medium between two electrodes [85].

The mass production of multi-walled nanotubes, product abundance, cheap raw materials, low production of by-products, and low energy consumption have made these pipes cheap. However, during their production, impurities may be created that require special operations to remove them, and even these reactions themselves change properties, such as the length of the pipes [86].

### 5.1. Mechanical Properties of Carbon Nanotubes on Dental Materials

Dental materials are subject to severe pressures in the mouth, so they must withstand the pressures of eating or certain diseases and all kinds of compressive, tensile, and shear forces. CNTs have different properties, such as high mechanical strength, low water resistance, and high absorption. Due to their unique structure, CNTs also have high tensile strength, equal to that of diamonds, so they can be used as implant coatings or even as its constituent material, causing extraordinary compressive, tensile, and shear properties in the implant [87].

### 5.2. Biocompatibility of Carbon Nanotubes

In the general implant placement process, the damaged tooth is removed, and the environment inside the gums is disinfected for the implant. After that, the healing process is performed and continues with the placement of the prosthesis. The healing process is known as fusion, in which bone cells adhere to the implant’s surface and lead to fusion. When the implant is placed into the bone, proteins first attach to its surface and mediate between bone cells and the implant surface. These proteins are involved in the processes of plaque adhesion, blood clotting, and inflammation [88]. CNT composites display biocompatibility similar to that of PLAGA(poly(lactic-co-glycolic acid)), a well-known FDA-approved biocompatible polymer. Thus, CNT is suitable for bone regeneration treatments and has a significant impact on the ability of clinicians to restore greater functional activity in injured bones [89].

### 5.3. Bone Growth and Proliferation in the Presence of Carbon Nanotubes

CNT is an excellent alternative to bone because bone cells can grow on it, it possesses high mechanical strength, and it mimics the microstructure of human trabecular bone [89]. A CNT study identified it as the ideal level for bone growth with the highest cell growth. The CNT also acts as a system enhancer, with the ability for various chemical groups to adhere to them and enhance their physical, chemical, and biological properties to improve bone growth.

**Figure 8:**
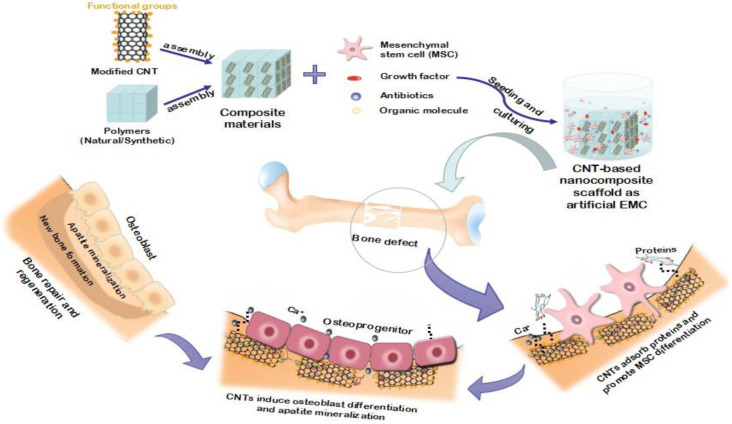
Diagram showing the role of carbon nanotubes (CNTs) as composite scaffolds in tissue engineering and regeneration [91].

Because CNT increases the surface roughness, it is used as a coating or in the structure of a layer (such as hydroxyapatite) and increases the adhesion of bone cells to the implant’s surface. The implant surface structure is an essential factor in implant welding because implants with a rough surface make it better welded to the bone. So far, most medical treatments for damaged bone have included replacing the lost bone with artificial materials. However, researchers have found that bone cells can grow and increase on a carbon nanotube scaffold with enough starting material. Tissue engineering promises that the placement of such scaffolds can regenerate the lost bone. Unlike many scaffolding materials, carbon nanotubes are not biodegradable and thus, create a conducive environment for cells to proliferate and deposit living bone material. Implanted carbon nanotube scaffolds can also enhance the mechanical properties of damaged bone tissue [88]. Carbon nanotubes, according to the researchers, can serve as such a framework. They used single-walled and multi-walled nanotubes to cultivate mouse bone cells, some of which were modified to collect electrically charged chemical groups. The proliferation of osteoblasts, cell shape, and the production of hydroxyapatite crystals during bone mineralization was used to evaluate each system’s biocompatibility. They discovered that the optimal environment for bone development is carbon nanotubes with a neutral electric charge [90]. Nanotube scaffolding growth was reduced when the charge of the nanotubes was changed to a net positive or negative charge. The findings showed that choosing single- or multi-walled nanotube scaffolds might affect the morphology of the cell. The role of carbon nanotubes (CNTs) as scaffold composites in bone tissue creation and regeneration is depicted in Figure 8.

### 5.4. The Antibacterial Activity of Nano Carbon

Infection in the surrounding tissue of the implant poses severe risks to the implant, and medical precautions are essential to prevent the activity of these bacteria. Inflammation around the gums is one of the most common diseases caused by bacteria. These bacteria lead to bone resorption around the implanted. Suggested solutions to fight bacteria are the following:

1. Levels of anti-microorganisms.
2. The manufacture of substances with antibacterial properties.

CNTs are inherently toxic; they prevent the growth of pathogenic bacteria and act as a practical surface on implants. CNTs also fight bacteria and kill them. In addition, CNTs perform drug delivery tasks very well. They can deliver antibiotics to any weak spot to aid in the healing process. The use of CNTs in cases such as titanium, zirconia, or carbon glass has caused implants’ surface load and surface structure to change [92].

Finally, CNT nanocomposites are designed to compensate for the weaknesses of commercial implants. CNTs have unique mechanical and biocompatibility properties that help us achieve our desired properties (mechanical stability, bone growth, and bacterial control).

However, such challenges as uniform composites production and a lack of understanding of their toxicity have limited their use. Current research focuses on the effect of carbon nanotubes on dental implants and their toxicity.

### 5.5. Application of Carbon Nanotubes in Dental Tissue

Because of its acceptable biocompatibility, similar to the main mineral part of bone tissue, which makes up 43 percent of its weight, and excellent ability to interact with and form grafts with natural bony tissue, hydroxyapatite (HAp) ceramic biomaterial has been used in bone grafting processes and orthopedic implant placement for more than a quarter of a century (with the chemical formula ((Cano (PO4) 6) (OH)) [93]. However, its weak fracture’s tensile strength and toughness relative to bone make it useless for main load-bearing devices in the skeletal system [94]. Due to their high length-to-diameter ratio and excellent mechanical properties, carbon nanotubes can make the hydroxyapatite matrix stronger and thicker without modifying biological activity and open a wide range of clinical uses of this material [91].

**Figure 9:**
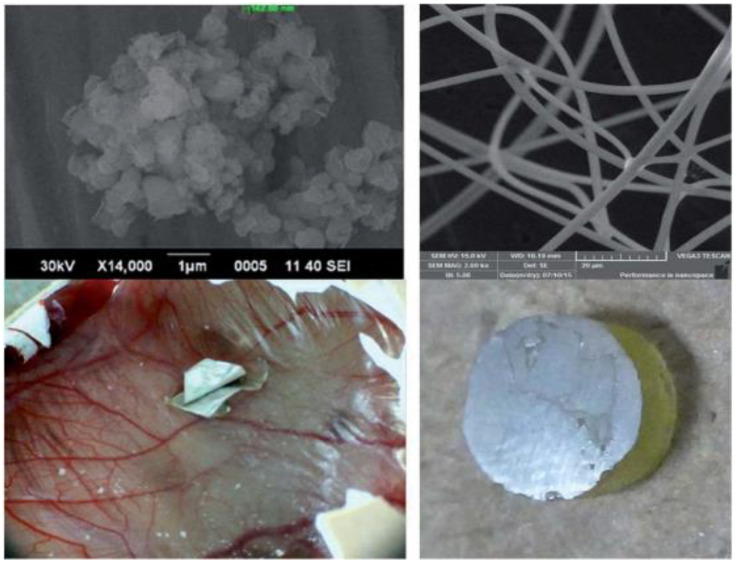
Electrospun hydroxyapatite/carbon nanotube fibers for biomedical/dental applications are manufactured and tested in vivo [95].

Khan et al. conducted a study to synthesize bioactive electrical filaments for biomedical and dental applications with more outstanding biocompatibility. The in situ precipitation of nanohydroxyapatite (nHA) was carried out at different concentrations (0.5%, 1%, 2%, 3%, and 5% by weight) of multi-walled carbon nanotubes. There were functions (MWCNT) by microwave irradiation (MW). HA/CNTs and CNTs were silanized with methacryloxypropyl trimethoxysilane (MPTS), mixed with polyvinyl alcohol (10% *w*/*v*) electrospun to make fibers. The biocompatibility of the two fibers was investigated, due to their effect on angiogenesis in the chicken chorion test (CAM). The mechanical properties showed a greater compressive strength of 3% loading HA/CNT (100.5_5.9 MPa) than other properties; the fracture behavior represents the dispersion of CNTs in the HA matrix. HA/CNT e-fibers showed higher angiogenesis than CNT fibers. Such HA/CNT electron filaments have revealed attractive targets for biocompatibility. Of course, due to the superior mechanical properties of CNT-reinforced composites, they can be used for dentin and periodontal reconstruction (Figure 9) [95].

**Figure 10:**
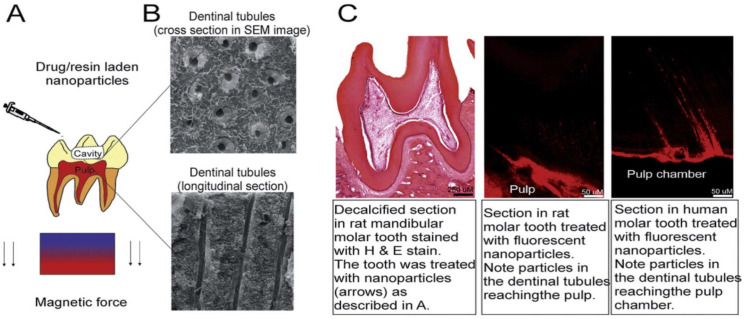
Description of the technology of conduction and delivery of drug-containing nanoparticles to the dental pulp. This technology exploits natural channels extending from the dentin to the pulp and magnetic force to deliver iron nanoparticles deep into the tooth structure. The technology was tested on rat molars and freshly extracted human teeth. It can be used to drug-load nanoparticles into the pulp or improve the bond strength of commercially available adhesive resins on the market in dentin (**A**) shows the surgery implantation of rhBMP-2 adsorbed MWCNT/CHI scaffolds into mouse subcutaneous muscular pocket. Optical microscope micrograph (**B**) shows regenerated bone tissue and a minor fraction of remaining MWCNT/CHI scaffold. Optical micrograph (**C**) shows a detail of regenerated bone tissue (collagen expressing cells, blue–green colored) after major disassembly of the MWCNT/CHI scaffold, surrounded by muscle tissue (pink colored). It is remarkable the well-limited interface between adjacent tissues (see black dash line). The remaining MWCNT/CHI scaffold (black colored) is pointed by black arrow [97].

Ji et al. developed a method for efficiently delivering treatment materials to the pulp based on NPs that can be actively guided by magnetic forces and travel through natural dentin channels (middle layer of the teeth). The treatment may reduce pulp discomfort and enhance dental adhesive penetration into the dentin. Compared to other treatment alternatives for damaged tooth pulp, the delivery mentioned above approach is less expensive, less painful, and less traumatic [96]. It is straightforward to comprehend and simple to use in therapeutic settings (Figure 10) [97,98].

## 6. Conclusions and Future Perspectives

One of the most fruitful sub-areas of nanomedicine and nanobiotechnology is biomedical applications of carbon-based nanomaterials. Studies on carbon-based biomaterials and their derivatives favor an incredible combination of physical and biological sciences for enhancing cellular interactions adhesion, proliferation, and osteogenesis differentiation.

Carbon-based nanoparticles offer excellent biocompatibility and biophysicochemical characteristics to guide bone regeneration and enhance osteoblast differentiation in dental tissue engineering. In addition, the use of carbon nanotubes (CNTs) and graphene oxide (GO) as nanocarriers for various drug delivery and cellular transport systems in the treatment of bone deformities and disorders was expanded, due to their enormous surface area, good biocompatibility, and stimulation.

Furthermore, carbon-based biomaterials offer tremendous potential to be used as novel biomaterials in dental tissue engineering. Carbon-based biomaterials are widely utilized for the reinforcement of dental implants, due to their outstanding mechanical properties, corrosion resistance, osteogenic properties, and antibacterial properties.

Despite the substantial advances and progress in applying carbon-based biomaterials for bone regeneration and repair, the experimental research still faces a number of difficulties in clinical application, which need to be overcome by the joint efforts of scholars and researchers worldwide. Firstly, follow-up investigations on carbon-based biomaterials’ low cytotoxicity and bioavailability will necessitate a full toxicological examination to ensure their safe clinical application. Biofunctionalized carbon-based composites and substrates were demonstrated to be biosafe and biocompatible for use in local bone scaffolds or implants; however, they should not loosen and migrate into the bloodstream, lungs, or abdominal cavity. For a better understanding of the complicated interactions between cells and materials, more emphasis should be directed to in vitro and in vivo biosafety investigations of carbon-based biomaterials. The most basic and effective method for reducing biotoxicity, increasing solubility, and combining various biological molecules is to functionalize carbon-based composites with covalent and non-covalent schemes; therefore, novel and creative technologies and strategies to modify these tubular structures must be studied and developed further. Second, carbon-based composites are particularly effective at promoting and inducing stem cell differentiation into specific lineages, particularly osteogenic differentiation. This could be attributed to the surface interaction between the cell membrane and the carbon-based biomaterial, which has a positive effect on cell behavior through absorption or repulsion of specific differentiation factors.

## Data Availability

Data available in a publicly accessible repository.
